# Platelet Function Testing Using Sonoclot and TEG6s as a Platelet Transfusion Prediction Tool in Open Heart Surgery

**DOI:** 10.7759/cureus.49131

**Published:** 2023-11-20

**Authors:** Tomoe Sato, Mitsutaka Edanaga, Michiaki Yamakage, Ryo Harada, Nobuyoshi Kawaharada

**Affiliations:** 1 Department of Anesthesiology, Sapporo Medical University School of Medicine, Sapporo, JPN; 2 Department of Cardiovascular Surgery, Shin-Sapporo Cardiology Hospital, Sapporo, JPN; 3 Department of Cardiovascular Surgery, Sapporo Medical University School of Medicine, Sapporo, JPN

**Keywords:** sonoclot, thromboelastography, cardiovascular surgery, transfusion, blood coagulation

## Abstract

Introduction

The point-of-care test (POCT) is useful for blood coagulation management during cardiovascular surgery. Although thromboelastography (TEG6s) has been reported to have targeted benefits for blood transfusion in cardiac surgery, Sonoclot analysis has not yet been fully validated. In this study, we evaluated the accuracy of Sonoclot, especially platelet function (PF) as a platelet concentrate (PC) transfusion parameter, compared to TEG6s in cardiovascular surgery.

Methods

This single-center, prospective, randomised trial was conducted at a university hospital. Forty-two adult patients who underwent elective cardiac surgery requiring cardiopulmonary bypass were included in this study between 2017 and 2021. The participants were randomly assigned to the Sonoclot (S) or Sonoclot and TEG6s (ST) groups. The amount of intraoperative PC was determined according to the POCT parameter values at the time of protamine administration. In addition, we investigated the correlation between PF parameters of POCT and platelet count at the end of surgery.

Results

There was no statistically significant difference in the intraoperative PC volume between the two groups. The Sonoclot PF parameter, PF, was moderately correlated with platelet count at the end of surgery (r=0.5449, p=0.009), and the TEG6s PF parameter showed a strong correlation with platelet count at the end of surgery (r=0.7744, p<0.001).

Conclusion

There was no statistically significant difference in platelet transfusion volume between the Sonoclot and TEG6s in this study. The correlation between the PF of the Sonoclot and platelet count was moderate. This study suggests that PF of Sonoclot may be a potentiating indicator of PF.

## Introduction

Blood coagulation disorders and loss after cardiopulmonary bypass (CPB) are serious complications of cardiovascular surgery. It has been reported that various factors can cause coagulopathy, including consumption of clotting factors and platelets, blood dilution, inadequate heparin reversal, and fibrinolysis [1-4]. A recent study has shown that major bleeding occurs in approximately 11% of patients undergoing cardiac surgery [5]. The use of allogeneic blood products has been identified as an independent risk factor for negative outcomes, such as renal dysfunction, stroke, infection, and mortality [6-7]. Other concerns regarding transfused blood products are that they are a limited resource of human origin. In Japan, the number of blood donors has declined by 31% over the past decade; therefore, a stable supply of blood has become a serious problem [8]. Globally, the proportion of young people in the transfused population is declining in the wake of the COVID-19 pandemic [9]. In addition, blood products for transfusion are expensive, and there is a particular concern about the financial pressure on Japan's medical insurance system. Therefore, the appropriate use of allogeneic blood products is required. However, among blood products, fresh-frozen plasma (FFP) is a troublesome product to use properly because guidelines for its use are not always followed [10-12]. Conventionally, the need for allogeneic blood products, such as FFP and platelet concentrate (PC), has been determined by referencing the results of standard laboratory tests (SLT) and surgical bleeding. The point-of-care test (POCT) provides results with a shorter turnaround time than SLT and enables the evaluation of platelet function (PF). A variety of POCT devices have been put into practical use, among which thromboelastography (TEG6s) has been examined for their utility in cardiovascular surgery in several studies and has been shown to be useful in reducing transfusion volume and predicting postoperative bleeding [13-14]. TEG6s platelet mapping (PM) was also reported to predict the change in PF from pre- to post-CPB [15]. On the contrary, Sonoclot (Sienco, Inc., Morrison, CO), which is an indicator of intraoperative transfusion decisions in cardiovascular surgery, has not yet been explored compared to TEG or rotational thromboelastometry (ROTEM) [14,16-18]. Sonoclot can measure PF by a cuvette consisting of a glass-bead activator. Previous reports have shown that Sonoclot might predict postoperative hemorrhage in cardiac surgery [19,20]. We have previously demonstrated the usefulness of Sonoclot as a transfusion strategy in cases of massive bleeding in trauma [21]. The operating cost of the Sonoclot is more efficient than that of other devices, which is a strong advantage in the Japanese insurance system [16]. In this study, we investigated the usefulness of Sonoclot as a transfusion parameter in cardiovascular surgery, especially in platelet transfusions, whose function is impaired in CPB. We hypothesize that Sonoclot may be useful as a guide to determine the need for PC transfusion.

## Materials and methods

Study design

This study was a single-center, prospective randomized study. Ethical approval for this study was obtained from the Institutional Review Board at Sapporo Medical University School of Medicine (292-21). The study was registered in the UMIN-CTR database (UMIN000027870). All participants provided written informed consent before enrollment in the study.

Participants and intervention

This study was conducted from 8 June 2017 to 31 March 2021. Participants aged 20-90 years, who were scheduled for elective adult cardiovascular surgery with CPB, were enrolled in this study. The participants were randomly divided into two groups, the Sonoclot group (Group S) and the Sonoclot with TEG6s group (Group ST) using a block randomization method (1:1 ratio). The exclusion criteria were as follows: no consent to participate in clinical research and mutual understanding. However, the use of oral antiplatelet agents and/or anticoagulants was not included in the exclusion criteria. If heparin was administered preoperatively, it was discontinued at least 6 hours before the start of surgery. In each case, 300 U/kg of heparin was administered before CPB. If activated clotting time (ACT) measured by Hemochron® response (Heiwa Busan, Tokyo, Japan) was less than 480, additional heparin (100 U/kg) was administered. Heparin levels were reversed after weaning from CPB with protamine until the ACT returned to almost pre-CPB values. Tranexamic acid (1000 mg), an antifibrinolytic drug, was routinely administered before and after CPB. Whole blood samples were obtained through a radial arterial catheter at three time points: 1) before induction of anesthesia, 2) 10 min after protamine administration, and 3) at the end of surgery. For Sonoclot analysis, a small amount (0.36 mL) of whole blood sample was placed into cuvettes consisting of a glass bead activator immediately after obtaining the blood samples. In Sonoclot, coagulation function was evaluated by clot rate (CR) and platelet function by PF. For TEG6s PM analysis, 300 μL of whole blood was collected into a heparin-filled tube and then applied to the cartridge within 30 min. In the TEG6s analysis, we used the platelet mapping method. We focused on the maximum amplitude in the kaolin with heparinase (HKH) assay (MA_HKH_), which reflects platelet-induced clot strength. At the end of the surgery, SLT was performed simultaneously with POCT. In the SLT, hemoglobin (Hb) concentration, fibrinogen concentration, platelet count, activated partial thromboplastin time (APTT), and partial thromboplastin time (PT) were measured. Postoperatively, the amount of bleeding from the drain was measured and recorded every few hours.

Management of blood products

**Figure 1:**
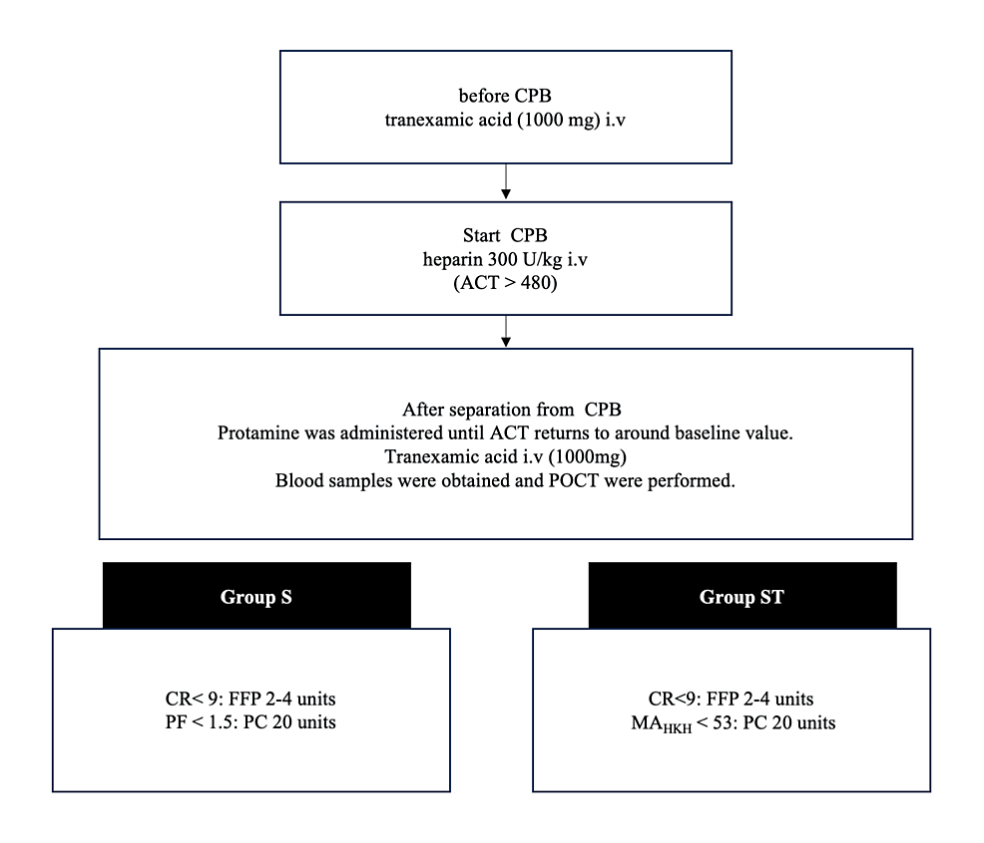
Coagulation and transfusion management algorithm during surgery If oozing in the surgical field was observed even after administering FFP and PC, additional FFP was used. CPB: cardiopulmonary bypass, ACT: activated clotting time, POCT: point-of-care testing, CR: clot rate, PF: platelet function; FFP: fresh frozen plasma, PC: platelet concentrate, MA_HKH_:_ _maximum amplitude in the kaolin with heparinase, S: Sonoclot, T: TEG6s

**Figure 2:**
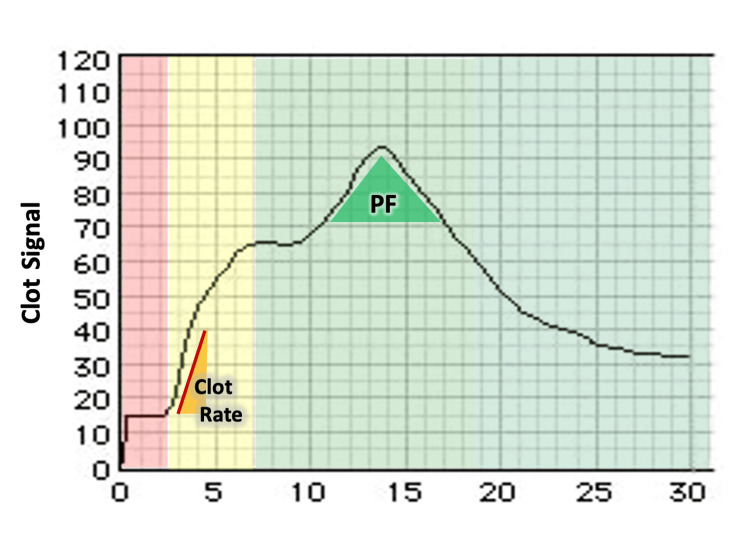
Schematic illustration of Sonoclot PF represents the retraction rate of platelets, which is calculated and quantified based on the rising rate of the slope and the angle of the apex. PF: platelet function

**Figure 3:**
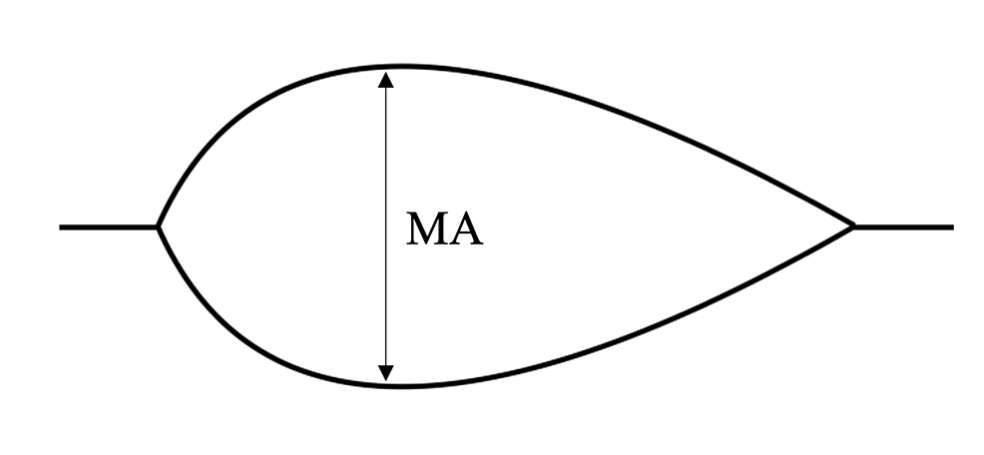
Schematic illustration of TEG6s MA reflects platelet-fibrin cross-link formation at the point of maximum clot strength (mm). MA: maximum amplitude

The blood management algorithm is shown in Figure 1. To compare the platelet function evaluation of the pure Sonoclot and TEG, the need for FFP in both groups was evaluated using only the CR of Sonoclot. If the CR was below the lower limit, two to four units of FFP were used depending on the decision of the anesthesiologist. When PF or MA_HKH_ was below the lower limit of the normal value, but there was no oozing in the surgical field, the decision whether to use PC or not was made after discussion between the anesthesiologist and surgeon, based on bleeding volume, CPB time, preoperative coagulation data, and POCT results. If oozing in the operative field continued after the administration of FFP and/or PC, additional FFP was administered. In both groups, red blood cell (RBC) transfusions were administered to keep Hb > 8.0 g/dl. A schematic illustration displaying the results of Sonoclot and TEG6s is shown in Figure 2 and Figure 3.

Outcomes

The primary outcome measure was the number of intraoperative PCs performed. Secondary outcomes were the intraoperative and postoperative amounts of RBC and FFP; intraoperative amount of salvaged blood volume; postoperative amount of RBC, FFP, and PC; intraoperative and postoperative bleeding volume; and correlation between platelet count and function at the end of surgery in both groups.

Statistical analysis

The sample size was based on a previous report [15]. A margin of non-inferiority was established to assess the non-inferiority of Sonoclot compared to TEG6s with respect to platelet transfusion volume. The preserved fraction was set to 0.5 as the conventional value. The effect of the standard intervention was set at 0.58 as the lower limit of 95% confidence intervals for the difference between the POCT group and the control group in the previous study [15]. Thus, the non-inferiority margin was calculated as 0.29. Considering an α of 0.05 and a power of 80%, the minimum required number of patients (one-sided test) was 38. Considering the dropout rate of 10%, 42 cases were enrolled in this study. Continuous dates are presented as means ± SD. Statistical analysis used a t-test and Fisher’s exact test for comparisons between groups. Correlations were tested by Spearman's correlation coefficient. A p value less than 0.05 was considered to represent statistical significance. All analyses were performed using GraphPad Prism version 8 software (MDF Co., Tokyo, Japan).

## Results

**Figure 4:**
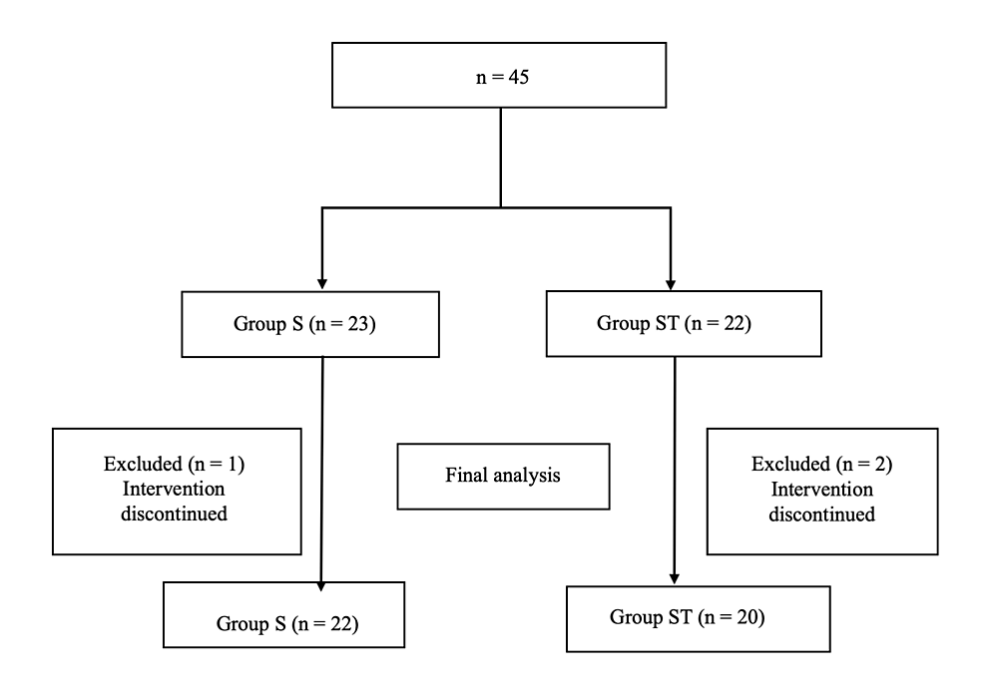
Flowchart of patient selection for group classification S: Sonoclot, T: TEG6s

**Figure 5:**
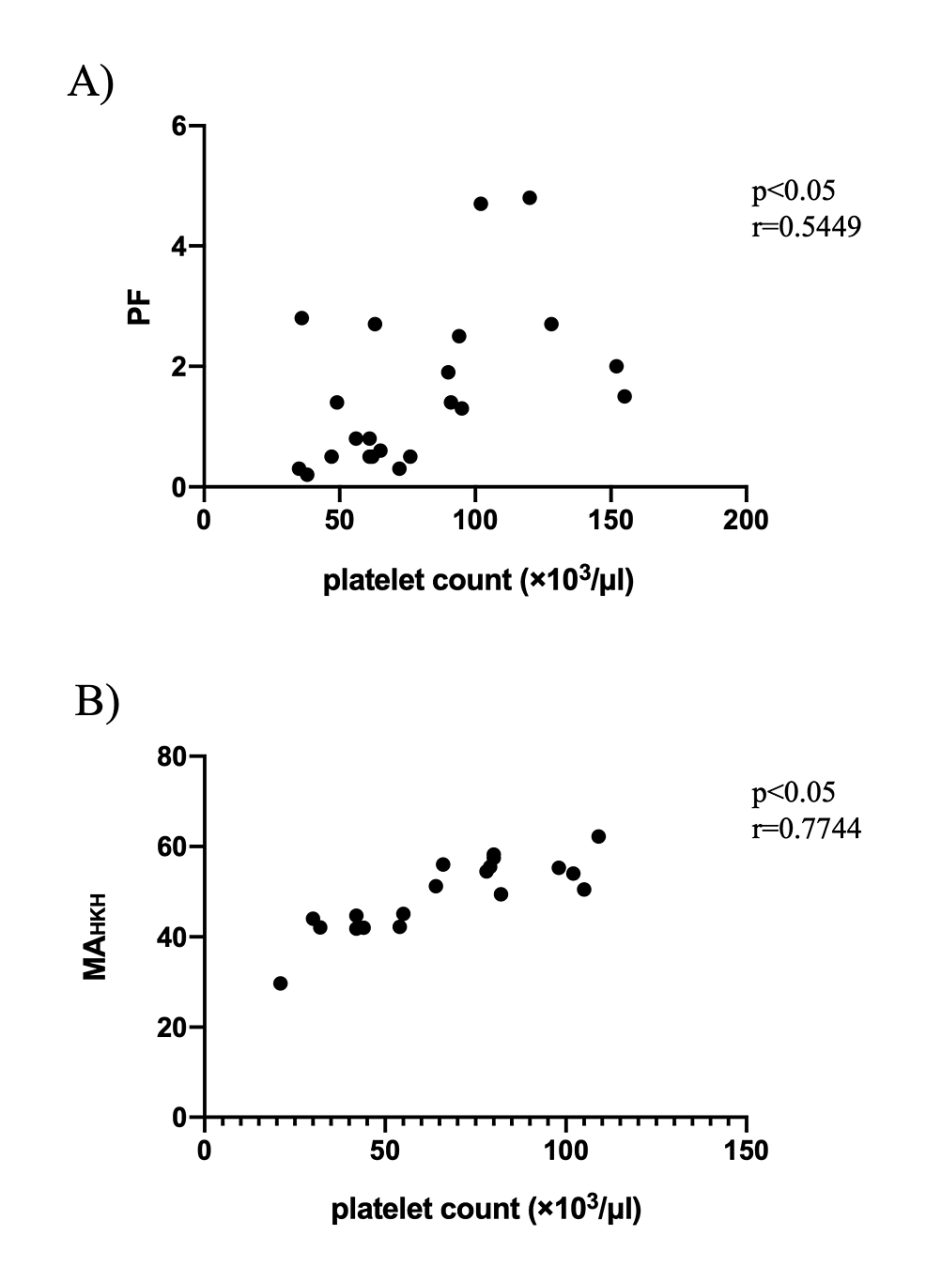
Correlation between platelet count and PF and platelet count and MAHKH at the end of surgery PF: platelet function; MA_HKH: _maximum amplitude in the kaolin with heparinase

A total of 45 patients were enrolled in the study. Three patients were excluded because they did not fulfill the inclusion criteria or because of the lack of data. Finally, 42 patients were analyzed (Figure 4). No statistically significant differences were observed between the two groups in terms of baseline characteristics (Table 1). Table 2 shows the pre- and postoperative SLT results. There were no statistically significant differences between the two groups. The intraoperative PC volume, which was the primary endpoint, was not significantly different between the two groups (Table 3). There were no significant differences in other parameters. The correlations between platelet count and PF, and platelet count and MA_HKH_ at the end of surgery are shown in Figure 5A and Figure 5B. The results showed a moderate correlation between platelet count and PF (r=0.5449, p=0.009) and a strong correlation between platelet count and MA_HKH_(r=0.7744, p<0.001). The intraoperative changes in the platelet count, PF of Sonoclot, and MA_HKH_ of TEG6s are shown in Supplemental Data (Appendix). Both parameters at two points (after protamine administration and postoperatively) were significantly lower than preoperative values. There was only one case of reoperation for bleeding in the Sonoclot group and no perioperative (within the first month after surgery) deaths in either group.

**Table 1:** Baseline characteristics The data are presented as means ± SD or the number of patients (percent). S: Sonoclot, T: TEG6s, M: male, F: female, BMI: body mass index, CABG: coronary artery bypass grafting, CPB: cardiopulmonary bypass

	Group S	Group ST	Difference means (95% CI)	P value
	N = 22	N = 20		
Sex (M: F)	13:9	11:9		0.27
Age (year)	67 ± 13	71 ± 9	4.0 (-3.3 to 11.3)	0.28
Height (cm)	160 ± 9	159 ± 10	-1.4 (-7.6 to 4.9)	0.66
Weight (kg)	62 ± 12	58 ± 12	-4.4 (-12.0 to 3.1)	0.24
BMI (kg/cm^2^)	24 ± 4	23 ± 4	-4.4 (-1.331 to 1.225)	0.97
Medicine				
Anticoagulant	3 (13)	7 (35)		0.10
Antiplatelet	4 (18)	5 (25)		0.59
Procedure				
CABG and valve surgery	2 (9)	2 (10)		
Single valve surgery	14 (63)	15 (75)		
Double valve surgery	1 (4)	1 (5)		
Aortic and valve surgery	2 (9)	1 (5)		
Other surgery	3 (13)	0 (0)		
CPB time (min)	176 ± 70	177 ± 66	0.64 (-42.3 to 43.6)	0.98
Anesthesia time (min)	415 ± 91	402 ± 76	-12.8 (-65.7 to 40.0)	0.63

**Table 2:** Standard laboratory test results at two time points S: Sonoclot, T: TEG6s, Hb: hemoglobin, aPTT: activated partial thromboplastin time, INR: international ratio

	Group S	Group ST	Difference means (95% CI)	p value
	N=22	N=20		
Platelete count [×10^4^/μl]				
preoperative	19.5 ± 6.9	17.4 ± 6.3	-2.1 (-6.3 to 2.1)	0.32
postoperative	7.9 ± 3.4	6.8 ± 2.7	-1.2 (-3.1 to 8.0)	0.24
Hb [g/dl]				
preoperative	12.8 ±1.6	12.6 ± 1.6	-0.17 (-1.2 to 0.83)	0.73
postoperative	9.9 ± 1.3	10.4 ± 1.5	0.49 (-3.3 to 11.3)	0.26
Fibrinogen [mg/dl]				
preoperative	317 ± 104	317 ± 68	0.16 (-57 to 58)	1
postoperative	166 ± 45	170 ±53	3.9 (-33 to 40)	0.83
aPTT [seconds]				
preoperative	31.3 ± 8.1	33.7 ± 9.8	2.4 (-3.2 to 8.0)	0.39
postoperative	49.1 ± 8.4	55.9 ± 19	6.8 (-4.1 to 17.6)	0.22
INR				
preoperative	1.03 ± 0.27	1.17 ± 0.51	0.14 (-0.11 to 0.39)	0.27
postoperative	1.4 ± 0.1	1.42 ± 0.4	0.06 (-0.13 to 0.26)	0.52

**Table 3:** The amount of intraoperative and postoperative transfused allogenic blood products and intraoperative salvaged blood volume, intraoperative and postoperative bleeding volume The data are presented as means ± SD or the number of patients (percent). RBC: red blood cell, FFP: fresh frozen plasma, PC: platelet concentrate

	Group S	Group ST	Difference means (95% CI)	p value
	N=22	N=20		
RBC (units)				
Intraoperative	3.8 ± 4.0	5.8 ± 4.4	2.0 (-0.7 to 4.6)	0.14
Postoperative	0.9 ± 2.5	1.6 ± 3.0	0.7 (-0.7 to 2.2)	0.31
FFP (units)				
Intraoperative	1.9 ± 1.9	2.4 ± 2.7	0.5 (-1.0 to 1.9)	0.50
Postoperative	1.9 ± 4.3	3.5 ± 4.0	1.6 (-0.6 to 3.8)	0.15
FFP transfusion	13 (59)	13 (65)		0.15
PC (units)				
Intraoperative	1.8 ± 5.7	2.1 ± 6.3	0.3 (-3.6 to 4.1)	0.88
Postoperative	2.3 ± 6.4	6.0 ± 9.4	3.7 (-1.2 to 8.6)	0.13
The number of PC transfused	2 (9)	2 (10)		0.92
Salvaged blood volume (ml)	591 ± 272	525 ± 320	-65 (-252 to 92)	0.48
Intraoperative bleeding volume (ml)	881 ± 597	935 ± 818	54 (-394 to 502)	0.81
Postoperative bleeding volume (ml)	313 ± 262	439 ± 303	126 (-53 to 306)	0.17

## Discussion

This is the first study to examine the potential of PF on Sonoclot as a platelet transfusion parameter. Although the correlation between the platelet count and PF was moderate, the intraoperative PC transfusion volume was similar in both groups. Intraoperative PF showed changes similar to MA_HKH_, suggesting that PF correctly reflects the deterioration of platelet function before and after CPB.

When preoperative risks, such as antiplatelet medications or low preoperative platelet counts, are not recognized, PC is usually not prepared during elective cardiovascular surgery in Japan. The cost of PC is high [22], and its necessity often depends on the degree of intraoperative bleeding and the discretion of the surgeons. Platelet count is an independent risk factor for massive hemorrhage in cardiovascular surgery [23]. However, the measurement of platelet counts requires considerable time to judge the results. Prothrombin time/international normalized ratio (PT-INR) reflects the activation of factors I, II, V, VII, IX, and X. APTT reflects the activation of coagulation factors I, II, V, VII, VIII, IX, X, XI, and XII and von Willebrand factor. However, PT-INR and APTT only assess the early stages of the coagulation system and do not accurately reflect the lack of coagulation factors in vivo. Therefore, it is difficult to determine from these tests the extent to which coagulation factor deficiency is involved in intraoperative bleeding tendency. In fact, a previous study showed that PT-INR > 1.5 and APTT > 1.5 times the basal value are not associated with hemorrhage [24]. However, platelet dysfunction is one of the main factors of bleeding, such as coagulopathy, extracorporeal circulation, and hyperfibrinolysis in Europe [3,25]. We can evaluate the parameter of POCT after 15 min. Although previous studies about the usefulness of TEG have been published, we cannot deny that there is limited research on the usefulness of Sonoclot during cardiovascular surgery. According to previous reports, the Sonoclot signature is thought to be effective in predicting postoperative hemorrhage in cardiovascular surgery [19,20,26]. In addition, Terada et al. reported that the CR of Sonoclot was a predictor of postoperative hemorrhage in cardiac surgery [27]. In the present study, the correlation between MA_HKH_ and platelet count at the end of surgery was strong, and the correlation between PF and platelet count was moderate. These results are generally consistent with the results of a previous study [27]. Postoperative bleeding volume tended to be higher in the S group, although this difference was not statistically significant. A previous study has shown that Sonoclot can predict massive bleeding after cardiovascular surgery, and when considered in conjunction with the result of this study, PF may reflect platelet function itself rather than platelet count [19]. In this study, our results at least showed that PC transfusion was not much in Group S. In this study, the cutoff values for PF and MA_HKH_ were the lower limit of normal for PF and MA provided by the manufacturer. The correlation between these parameters after protamine administration was strong (coefficient r=0.882; date is not shown). The results suggest that Sonoclot reflects platelet function to the same degree as TEG6s. Postoperative bleeding volume tended to be higher in the S group, although this difference was not statistically significant.

We conducted this study because we were concerned that blood transfusion resources would become scarce in Japan’s super-aging society. In Japan, there is no insurance support for the use of blood components or processed coagulation factor concentrates, such as fibrinogen and factor Ⅶ concentrates, in cardiac surgery. Thus, although several techniques are available to reduce the use of transfusion products, such as tranexamic acid, cell salvage, retrograde autoprime, and normal blood dilution, options other than autologous blood products for coagulation disorders in Japanese cardiovascular surgery are very limited. Under these circumstances, we believe that the POCT containing Sonoclot can be used to promote the efficient use of transfused blood products.

Our study had some limitations. First, our study included different types of surgeries performed at a single center. Second, we did not exclude patients who continued to receive antifibrinolytic or antiplatelet agents. The effects of these drugs, particularly the amount of blood transfused and intraoperative bleeding, may have affected the results of this study. Third, this was a single-center, not a multicenter study.

## Conclusions

In conclusion, Sonoclot might be non-inferior for the parameter of PC transfusion compared with TEG6s in cardiovascular surgery. Further large-scale studies are needed to establish the validity of Sonoclot as a platelet transfusion parameter.

## Figures and Tables

**Figure 6:**
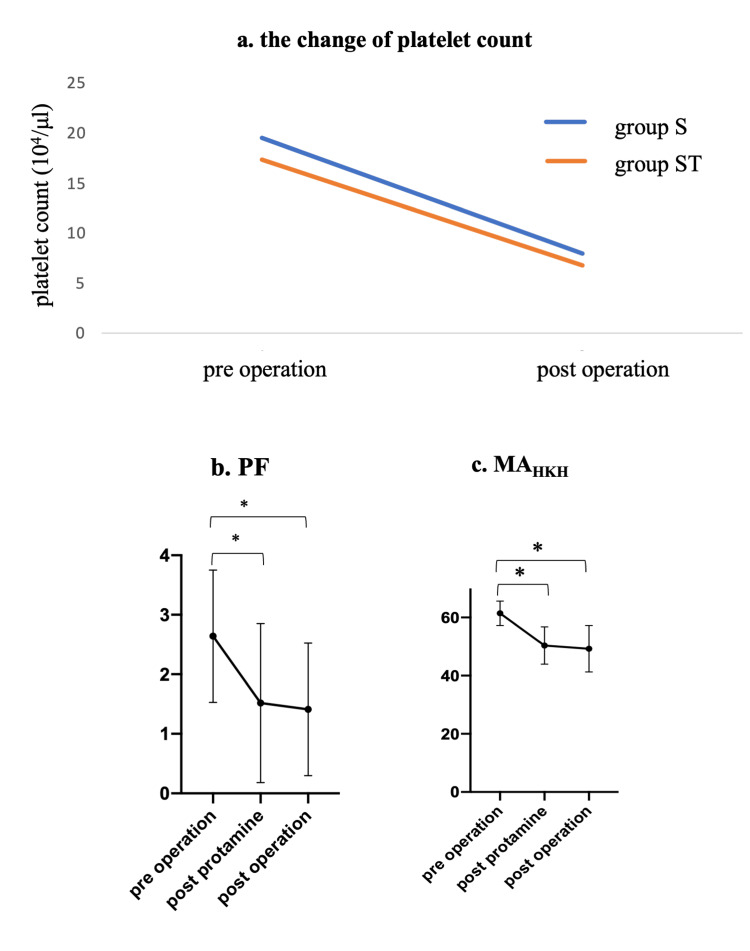
Supplemental data. The changes in platelet count(a) and platelet function of Sonoclot and TEG6s (b, c) through surgery S: Sonoclot, T: TEG6s, PF: platelet function, MA_HKH_: maximum amplitude in the kaolin with heparinase
